# Oxygen Consumption Characteristics in 3D Constructs Depend on Cell Density

**DOI:** 10.3389/fbioe.2019.00251

**Published:** 2019-10-10

**Authors:** Chiara Magliaro, Giorgio Mattei, Flavio Iacoangeli, Alessandro Corti, Vincenzo Piemonte, Arti Ahluwalia

**Affiliations:** ^1^Research Center “E. Piaggio”, University of Pisa, Pisa, Italy; ^2^Department of Information Engineering, University of Pisa, Pisa, Italy; ^3^Department of Engineering, University “Campus Bio-medico” of Rome, Rome, Italy; ^4^Department of Traslational Research and New Technologies in Medicine and Surgery, University of Pisa, Pisa, Italy

**Keywords:** oxygen consumption rate, reaction, diffusion, Michaelis-Menten, scaffold, 3D cell culture

## Abstract

Oxygen is not only crucial for cell survival but also a determinant for cell fate and function. However, the supply of oxygen and other nutrients as well as the removal of toxic waste products often limit cell viability in 3-dimensional (3D) engineered tissues. The aim of this study was to determine the oxygen consumption characteristics of 3D constructs as a function of their cell density. The oxygen concentration was measured at the base of hepatocyte laden constructs and a tightly controlled experimental and analytical framework was used to reduce the system geometry to a single coordinate and enable the precise identification of initial and boundary conditions. Then dynamic process modeling was used to fit the measured oxygen vs. time profiles to a reaction and diffusion model. We show that oxygen consumption rates are well-described by Michaelis-Menten kinetics. However, the reaction parameters are not literature constants but depend on the cell density. Moreover, the average cellular oxygen consumption rate (or OCR) also varies with density. We discuss why the OCR of cells is often misinterpreted and erroneously reported, particularly in the case of 3D tissues and scaffolds.

## Introduction

Engineered tissues have many applications, ranging from *in-vitro* models of human pathophysiology to platforms for drugs and treatment testing (Lee et al., 2005), to providing an alternative to the shortage of human donor organs for transplants (Mattei et al., 2017b, 2018). However, the supply of oxygen and other nutrients as well as the removal of toxic waste products, which typically occur via passive diffusion in *in-vitro* cellular constructs, often limit cell viability in thick (millimeter-sized) engineered tissues (Mattei et al., 2014). The typical diffusion limit of cell-rich tissues, such as skeletal muscle or liver, is considered to be of ~200 μm, which defines the side of the so-called *functional unit*, i.e., the smallest autonomous organ cubic voxel which can survive in the absence of blood vessels (Helmlinger et al., 1997; Mattei et al., 2014). *In-vivo*, nutrient supply and waste removal for thicker tissues are enhanced by convection through the vascular system, which is typically absent *in-vitro* (Folkman and Moscona, 1978; Rivron et al., 2008). Oxygen is considered the limiting factor when culturing three-dimensional (3D) cell constructs *in-vitro*, especially in the case of non-porous systems where its mass transport relies only on gradient-driven passive diffusion. The reason for this is its poor solubility in culture media (typically ~0.2 mM, when atmospheric oxygen is used), making it difficult to provide a sufficient resource supply to cells in the core of the engineered 3D constructs. In fact, although oxygen is typically consumed at a similar molar rate per cell as glucose and has a ~4-fold higher diffusion coefficient in aqueous media, this is more than offset by its very low solubility with respect to that of glucose, which results in an O_2_ availability of about 0.05–0.2 mM under physiologically relevant conditions, vs. 3–15 mM of glucose (Martin and Vermette, 2005). Moreover, using pure oxygen instead of air or increasing gas pressure without an appropriate carrier such as hemoglobin to increase oxygen concentration in the culture medium has been shown to induce the presence of free radicals, which are cytotoxic (Freshney, 2015). For this reason, the culture medium is often re-circulated and re-oxygenated by passing through an in-line gas exchanger (Mattei et al., 2017a; Ahluwalia et al., 2018). As oxygen is not only crucial for cell survival but also a determinant for cell fate and function, a better understanding of its diffusion and consumption in *in-vitro* constructs is critical to enable improvements in tissue engineering.

In most studies on oxygen utilization, the parameter reported is the *average* cellular oxygen consumption rate or *OCR*, expressed as [mol·cell^−1^ · s^−1^], typically estimated by measuring the *overall* oxygen consumption rate (in mol·s^−1^) in the system and dividing by the total number of cells present. The range of *OCR* is usually between 1 × 10^−16^ and 1 × 10^−18^ mol · cell^−1^ · s^−1^ (or 100 to 1 amol·cell^−1^·s^−1^) (Wagner et al., 2011), but the fact that it is an average consumption rate per cell and not an absolute value is often overlooked. Noticeably, the value varies according to experimental conditions and cell type; for instance, the *OCR* measured for a 2D monolayer of hepatocytes (Smith et al., 1996; Balis et al., 1999)—which are known to have oxygen consumption rates of about 10–100 times higher than most other cell types (Cho et al., 2007)—appears to be an order of magnitude greater than that measured for 3D cultures (Shatford et al., 1992; Nyberg et al., 1993, 1994; Sielaff et al., 1997). Moreover, hepatocyte *OCR* seems to decrease with increasing cell density in 3D cell cultures. Patzer attributed this phenomenon to the fact that denser cultures better approximate the physiological density of native liver, thus cells are less metabolically “stressed” (Patzer, 2004). Interestingly, cells *in-vivo* generally have much lower *OCRs* than their *in-vitro* counterparts (Glazier, 2015; Ahluwalia, 2017; Magliaro et al., 2019).

Computational fluid dynamic (CFD) models have been largely used to investigate whether cell culture parameters (such as culture medium flow) and configuration (e.g., volume and geometry of both fluidic compartment and cell construct) are appropriate for a given cell type. The models enable quantitative estimates of nutrient supply and waste removal capacity as well as flow-induced shear stress. Several mathematical models which describe oxygen consumption and transport in engineered tissues have been published and the resulting spatial gradients of oxygen concentration have been correlated with either cell density (Lewis et al., 2005; Demol et al., 2011), viability (Radisic et al., 2006; Cheema et al., 2012), organization (Malda et al., 2004; Brown et al., 2007), metabolism (Zhou et al., 2008) or growth factor expression (Mac Gabhann et al., 2007; Cheema et al., 2008).

In most of these models, the volumetric oxygen consumption rate within the cell construct (*R* in [mol·m^−3^·s^−1^]) is described by the well-known Michaelis-Menten law (Equation 1):

(1) $$\begin{array}{l} R\left(x,y,z,t\right)=\frac{V_{\max}\;\cdot \;C\left(x,y,z,t\right)}{K_{m}\;+\;C\left(x,y,z,t\right)} \end{array}$$

Note that *R* depends on the local oxygen concentration (*C*) that is generally a function of both space (lateral dimensions *x* and *y*, height *z*) and time (*t*), i.e., *C*(*x, y, z, t*). *V*_max_ and *K*_*m*_ are respectively, the maximum volumetric oxygen consumption rate and the Michaelis-Menten constant (corresponding to the oxygen concentration at which consumption drops to half of its maximum and expressed in [mol·m^−3^]). The terms *V*_max_ and *K*_*m*_ are considered as constants for a given system. Since *C* is the only variable, the cellular oxygen consumption rate is “adaptive” only with respect to the local oxygen concentration.

Using the above equation, Ahluwalia (2017) showed that the *OCR* is identical for each cell plated in a monolayer as they are all the same distance from the oxygen supply and hence they perceive the same oxygen concentration at any instant (i.e., *C* is the same for all x and y, while z is constant). Conversely, in 3D constructs as *in-vivo*, cells close to the media or to a capillary perceive high levels of oxygen, and so consume at a higher rate, while those further away perceive lower levels and hence consume at a lower rate. Thus, the average oxygen consumption rate per cell is lower in 3D than in 2D.

Besides cell type and 2 or 3D configuration, the oxygen consumption characteristics may also depend on other factors such as the cell density or even variations in intrinsic consumptions constants such as *K*_*m*_. In this study we therefore investigated oxygen utilization in 3D constructs as a function of cell density (ρ_*cell*_ in [cells·m^−3^]) through a combined experimental-computational approach. In particular, we focused on 3D hydrogels laden with hepatocytes at different densities. Parameters typically considered as constants in mathematical models of oxygen transport (i.e., diffusion coefficient, *D*) and consumption (i.e., *V*_max_ and *K*_*m*_) were treated as variables in this study and derived by fitting experimental oxygen measurements with a time-dependent computational model.

## Materials and Methods

### Oxygen Sensing Setup

Experiments were performed in a commercial polydimethylsiloxane (PDMS) bioreactor (LiveBox1, IVTech s.r.l., Italy) featured with a glass top and bottom, which provides an optically transparent cylindrical cell culture chamber (15 mm diameter−16 mm height). A 15 mm diameter RedEye® Fospor oxygen sensor patch (Ocean Optics, Germany) was attached to the inner side of bioreactor glass bottom to continuously monitor the oxygen concentration at the base of the cell culture chamber. This sensor uses dynamic fluorescence quenching of a ruthenium complex in a sol-gel and allows for non-invasive/non-destructive measurements through a transparent material without consuming oxygen (measurement range: 0–21% O_2_; accuracy: ±0.01% O_2_; response time: <5 seconds). Real time oxygen measurements were made by reading oxygen sensor fluorescence with a RE-BIFBORO-2 optical fiber (Ocean Optics) coupled to the outside of the bioreactor glass bottom and connected to a NEOFOX-GT phase fluorometer. Partial oxygen pressure was obtained from fluorescence through the Stern-Volmer equation and then converted to a concentration using Henry's Law (Curcio et al., 2010). A two-point calibration curve was obtained first by reading the fluorescence after pipetting 500 μL of fresh Eagles Minimal Essential Medium (EMEM) culture medium into the bioreactor culture chamber. This corresponds to 20% oxygen partial pressure or a concentration of 0.2 mol·m^−3^ (Curcio et al., 2010). The second point was obtained by adding 500 μL of EMEM containing 1% w/v sodium bisulfite (NaHSO3, Sigma-Aldrich), which establishes the point at 0% O_2_ partial pressure, i.e., 0 mol·m^−3^ oxygen concentration (Tremper et al., 1986).

### Cell Source

Human hepatoma HepG2 cells were purchased from ICLC (Genova, Italy) and cultured according to the supplier's instructions. Cells were grown in EMEM with Earle's Balanced Salts (EBSS) supplemented with 10% v/v fetal bovine serum, 2 mM glutamine and 1% v/v non-essential amino acids in a 37°C/5% CO_2_ humidified incubator. A trypsin/EDTA solution was used to detach confluent cultures. The number of viable cells was assessed by manual cell count in a Burker chamber with the trypan blue exclusion test. After the count, cells were centrifuged (300 × g, 5 min, room temperature) and resuspended in media at the required cell density.

### Cell-Laden Hydrogel Construct

**Figure 1:**
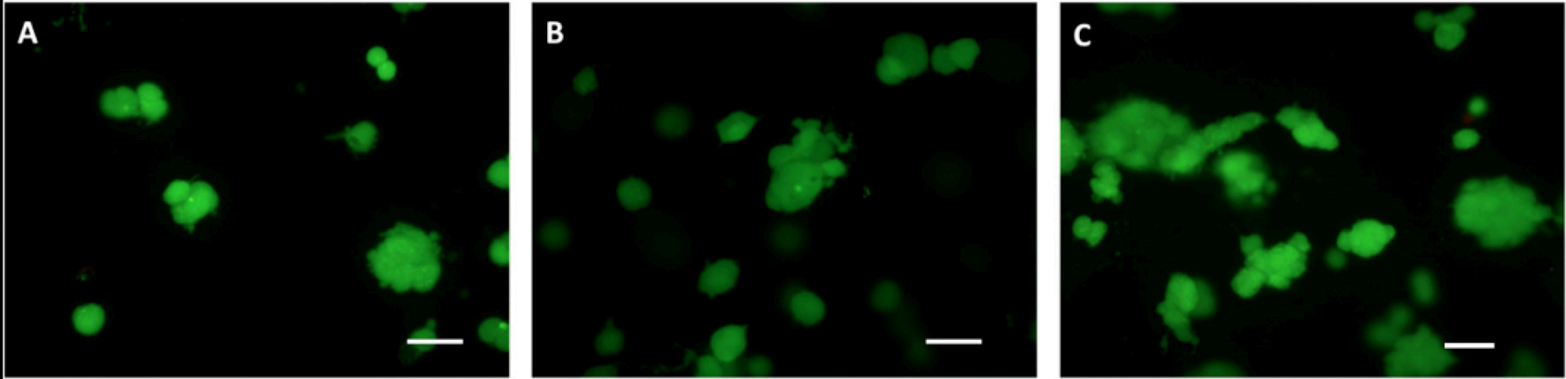
HepG2 cells encapsulated in the gels, stained with Live-Dead. Cell densities: **(A)** 0.5·10^12^, **(B)** 1·10^12^, and **(C)** 5·10^12^ cells·m^−3^. Scale bar: 50 μm.

Hydrogels laden with HepG2 cells at different densities (i.e., 0.5·10^12^, 1·10^12^, and 5·10^12^ cells·m^−3^) were prepared using a sterile 3 mg/mL collagen solution from bovine skin (C4243, Sigma-Aldrich). The collagen solution was mixed in an 8:1 volume ratio with M199 10x medium (Sigma) containing HepG2 cells at 9 times the final desired density. A 1N NaOH solution was added drop-wise to adjust the pre-gel solution pH to 7.4. The preparation was carried out on ice to prevent collagen gelation. Then, 220 μL of the cell-containing pre-gel solution were pipetted onto the bioreactor glass bottom (previously covered with the oxygen sensor patch) and incubated for 1 h at 37°C to allow the collagen polymerization, obtaining a 1.25 mm thick cell-laden collagen construct. All samples were tested immediately after polymerization in order to guarantee a nearly constant initial value of ambient oxygen concentration (20%) throughout their thickness. A sample of the gels was also stained with Live-Dead®Viability/Cytotoxicity Kit, for mammalian cells (L3224 - ThermoFisher, Waltham, MA USA). The solution was added to sample media according to manufacturer's instructions and incubated for 20 min at room temperature. Gels were then transferred onto glass microscope slides and imaged with an Olympus IX81 inverted microscope with a 10x objective. Figure 1 shows the cells within the hydrogel scaffolds at the different cell densities investigated.

### Oxygen Consumption Measurements

Oxygen measurements were performed by placing the setup inside a 37°C/5% CO_2_ humidified incubator. At the beginning of the experiment (*t* = 0), hydrogel constructs polymerised and maintained in the 20% O_2_ incubator (thus containing 20% O_2_ concentration, in equilibrium with the overlying culture medium) were exposed to a step change in the O_2_ concentration of their overlying culture medium. Hypoxic culture conditions were obtained by replacing the 500 μL of EMEM culture medium with the EMEM containing 1% w/v sodium bisulfite. Even though the medium was the same as that used for sensor calibration, we found that—in presence of the hydrogel—the oxygen concentration in the overlying medium was maintained at a constant level of 1·10^−2^ mol·m^−3^, instead of the expected 0 mol·m^−3^, during the whole experiment (i.e., up to 3 h, measured at different times and positions with a commercial Ocean Optics needle oxygen sensor; data not shown). Experiments at the three different cell densities were performed in triplicate while keeping all the other experimental parameters (e.g., oxygen sensing setup, hydrogel composition, volume and thickness, culture medium composition, acquisition time, initial and boundary conditions) unaltered. A total of 9 collagen hydrogels were analyzed.

## Mathematical Model

### Geometry

**Figure 2:**
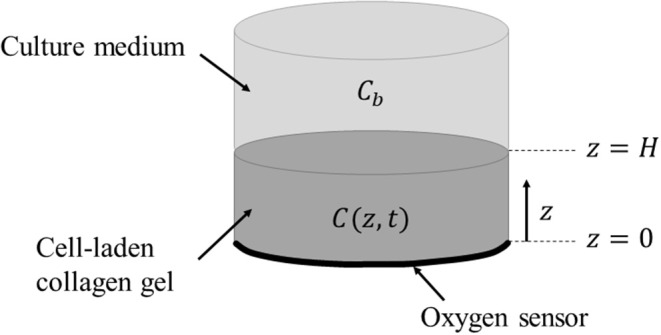
Schematic representation of the modeled system consisting of a cell-laden collagen hydrogel polymerised inside the bioreactor chamber on top of the O_2_ sensor. The gel is covered with culture medium with a uniform and time-invariant oxygen concentration (***C***_***b***_). The oxygen sensor enables real-time, non-invasive measurement of O_2_ concentration at *z* = 0 by reading fluorescence via an external optical fiber.

Figure 2 shows the geometry of the mathematical model describing the oxygen transport throughout the cell-laden construct detailed in section Materials and Methods. The oxygen concentration within the collagen gel of thickness H = 1.25 mm and radius of 7.5 mm is a function of both the vertical coordinate and time, *C*(*z, t*). The bulk environment (i.e., culture medium) directly above the collagen gel is assumed to maintain a uniform and time-invariant oxygen concentration, *C*(*H, t*) = *C*_*b*_, representing the hypoxic boundary.

### Oxygen Diffusion and Consumption Rate

Given the cylindrical symmetry of the system investigated, mass transfer throughout the cell-laden hydrogel occurs by passive diffusion in the axial direction only, since oxygen concentration is assumed to be independent of the radial and polar dimensions. The mass balance of oxygen in the cell-laden hydrogel is then described by the Fick's second law with a reaction term:

(2) $$\begin{array}{l} \frac{\partial C\left(z,t\right)}{\partial t}=D\frac{\partial^{2}C\left(z,t\right)}{{\partial z}^{2}}-R\left(z,t\right) \end{array}$$

where *C* [mol^.^m^−3^] is the concentration of oxygen as a function of space and time, *D* [m^2^·s^−1^] is the diffusion coefficient of oxygen in the gel (assumed to be spatially homogeneous), and *R* [mol O_2_ consumed·m^−3^·s^−1^] is the cellular oxygen consumption rate per unit volume of the construct, assumed to be homogeneously populated with cells. Changes in cell number due to proliferation or death during the time scale of the cellular experiments (<1 h) were neglected due to the short duration of the experiment. Cellular oxygen consumption was modeled using the Michaelis-Menten kinetics (Mattei et al., 2014) (Equation 1), by substituting _*V*_max_ = ρ*cell*_·*sOCR* and considering only oxygen concentration variations in time and height, i.e., *C*(*z, t*), obtaining:

(3) $$\begin{array}{l} \frac{\partial C\left(z,t\right)}{\partial t}=D\frac{\partial^{2}C\left(z,t\right)}{{\partial z}^{2}}-\;\frac{\rho_{cell}\;\cdot \;sOCR\;\cdot \;C\left(z,\;t\right)}{K_{m}\;+\;C\left(z,t\right)} \end{array}$$

In Equation (3), the *sOCR* (mol·cell^−1^·s^−1^) is the maximum rate of oxygen consumption per single cell. At high oxygen concentrations (i.e., when *C*(*z, t*) ≫ *K*_*m*_), the system is saturated and the rate of reaction is maximal, *R*(*z, t*) = _*V*_max_ = ρ*cell*_ · *sOCR*.

As pointed out in the introduction, most studies on oxygen utilization report the *average* cellular oxygen consumption rate (*OCR*, expressed as [mol · cell^−1^·s^−1^]), a time-dependent parameter which can be expressed as the volumetric average of the local *OCR* (dependent on both time and space) within the cell construct of volume *V* (Equation 4):

(4) $$\begin{array}{l} OCR\left(t\right)=\frac{1}{V\;\cdot \;\rho_{cell}}∭\frac{\rho_{cell}\;\cdot \;sOCR\;\cdot \;C\left(x,y,z,\;t\right)}{K_{m}\;+\;C\left(x,y,z,t\right)}\partial x\;\partial y\;\partial z \end{array}$$

Given the cylindrical symmetry of the problem investigated in this work, Equation (4) simplifies to:

(5) $$\begin{array}{l} OCR\left(t\right)=\frac{\pi R^{2}}{V\;\cdot \;\rho_{cell}}\int \frac{\rho_{cell}\;\cdot \;sOCR\;\cdot \;C\left(z,\;t\right)}{K_{m}+C\left(z,t\right)}\partial z=\\ \;\frac{1}{H}\int \frac{sOCR\;\cdot \;C\left(z,\;t\right)}{K_{m}+C\left(z,t\right)}\partial z \end{array}$$

Notably, at high oxygen concentrations, where *C*(*z, t*) ≫ *K*_*m*_ ,the *OCR* tends to the *sOCR*.

### Boundary and Initial Conditions

In each experiment, oxygen concentration measurements started when the EMEM culture medium overlying the hydrogel construct was replaced with that containing 1% w/v sodium bisulfite, setting the experimental time zero (*t* = 0). Atmospheric air contains 20.9% oxygen, which reduces to 20% O_2_ in cell culture incubators due to the presence of 5% CO_2_. Because all the hydrogels were prepared under ambient conditions and then polymerised and maintained in a 20% O_2_ incubator before experiments, the initial concentration of oxygen in the gel was assumed to be spatially uniform and equal to *C*(*z, t* = 0) = *C*_0_ = 0.2 mol·m^−3^. Conversely, the O_2_ concentration in the overlying culture medium was experimentally found to be *C*_*b*_ = 1% O_2_ (section Oxygen Consumption Measurements), thus giving the boundary condition *C*(*z* = *H, t*) = 1·10^−2^ mol·m^−3^. No flux boundary conditions were used for all boundaries except the top surface of the gel (*z* = H, which was held constant at *C*_*b*_), implying that:

(6) $$\begin{array}{l} \frac{\partial C\left(z\;=\;0,t\right)}{\partial z}=0 \end{array}$$

### Non-dimensionalisation of the Governing Equation

The governing diffusion-reaction equation (Equation 2) was rendered non-dimensional by introducing the following group of variables:

(7) $$\begin{array}{lll} \Theta & = & \frac{C}{C_{0}} \end{array}$$

(8) $$\begin{array}{lll} \tau & = & \frac{t\;\cdot \;D}{H^{2}} \end{array}$$

(9) $$\begin{array}{lll} \zeta & = & \frac{z}{H} \end{array}$$

where Θ, τ and ζ represent dimensionless concentration, time, and length, respectively.

In case of combined diffusion and reaction phenomena it is useful to introduce the Thiele modulus, a dimensionless parameter representing the ratio between the characteristic reaction rate and diffusion rate of a given species throughout the hydrogel construct, defined as (Cheng et al., 2009):

(10) $$\begin{array}{l} \phi =H\sqrt{\frac{sOCR\;\cdot \;\rho_{cell}}{D\;\cdot \;K_{m}}} \end{array}$$

Equation (3) can then be rewritten in the following dimensionless form:

(11) $$\begin{array}{l} \frac{\partial \Theta }{\partial \tau }=\frac{\partial^{2}\Theta }{{\partial \zeta }^{2}}-\phi^{2}\frac{\Theta }{1\;+\;\frac{\Theta }{K_{m}}} \end{array}$$

which is subjected to the initial and boundary conditions below:

(12) $$\begin{array}{l} \Theta \left(0,\;\zeta \right)=1 \end{array}$$

(13) $$\begin{array}{l} \Theta \left(\tau ,\;1\right)=\frac{C_{b}}{C_{0}} \end{array}$$

(14) $$\begin{array}{l} \frac{\partial \Theta \left(\tau ,\;0\right)}{\partial \zeta }=0 \end{array}$$

A summary of all the model parameters is presented in Table 1.

**Table 1:** Summary of model parameters.

**Parameter**	**Definition**	**Units**
*C*	Dissolved oxygen concentration in the hydrogel construct	% or mol·m^−3^
*z*	Vertical distance from the base of the hydrogel construct	m
*t*	Culture time under hypoxic conditions	s
*D*	Oxygen diffusion coefficient in the hydrogel construct	m^2^ · s^−1^
*V*_max_	Maximum oxygen uptake	mol·m^−3^ · s^−1^
s*OCR*	Single cell maximum oxygen consumption rate	mol·cell^−1^ · s^−1^
*K*_*m*_	Half-maximum rate oxygen concentration	% or mol·m^−3^
Θ	Dimensionless oxygen concentration	–
τ	Dimensionless time	–
ζ	Dimensionless length	–
ϕ	Thiele modulus	–

### Parameter Estimation

The dynamic process modeling software gPROMS (generalized PRocess Modeling System; PSE; London, UK) was used to fit the oxygen concentrations measured at the base of the hydrogel to the non-dimensional model equations shown in section Non-dimensionalisation of the Governing Equation. The unknown parameters *D*, *V*_max_ and *K*_*m*_ were estimated by fitting experimental data obtained from cell-laden hydrogels at the three different volumetric cell densities (i.e., ρ_1_ = 0.5 · 10^12^ cells·m^−3^, ρ_2_ = 1 · 10^12^ cells·m^−3^ and ρ_−3_ = 5 · 10^12^ cells·m^−3^). The *goodness of fit* was assessed by computing the coefficient of variation (*CV*) for each estimated parameter, defined as the ratio between the standard deviation and the mean of the estimate. In particular, estimates with low values of *CV* denote a good fit.

## Results

### Experimental Oxygen Concentration Profiles, Fitting Results, and Estimated Parameters

**Figure 3:**
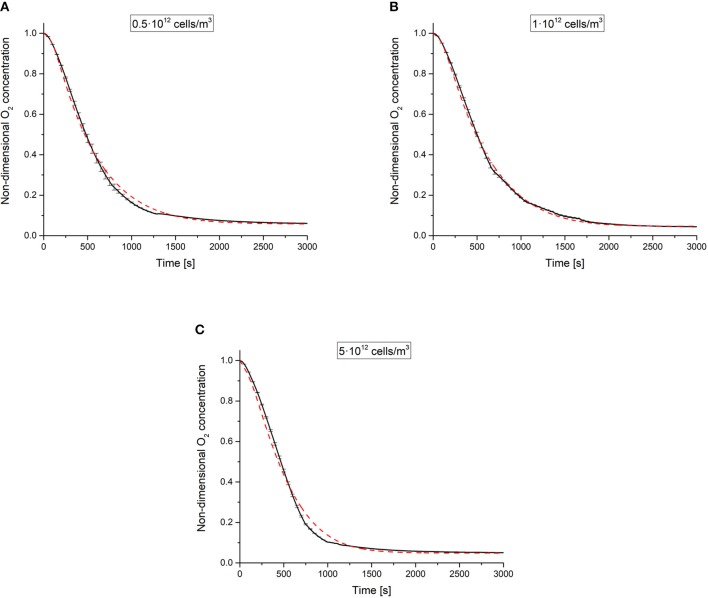
Experimental non-dimensional oxygen concentrations vs. time (black lines, average data from *n* = 3 independent experiments per each cell density investigated) and respective model estimations (dashed red lines) obtained for different cell densities: **(A)** 0.5·10^12^, **(B)** 1·10^12^, and **(C)** 5·10^12^ cells·m^−3^. Error bars—shown every 50 s for the sake of clarity—denote standard deviations of experimental data.

Figure 3 shows the experimental non-dimensional oxygen concentrations measured at the base of the 3 different cell-laden hydrogel constructs over time, i.e., Θ(*z* = 0, *t*). The computed Θ(*z* = 0, *t*) temporal profiles—derived by fitting experimental data obtained at different cell densities to the model described in section Mathematical Model—are shown in the same figure along with their respective measured profiles. A good fit was obtained for all the 3 different cell-laden constructs investigated. Estimated parameters and the corresponding *CV* values are summarized in Table 2. The parameters identified in this study lie within the values reported in the literature (Patzer, 2004; Wagner et al., 2011).

**Table 2:** Estimated oxygen diffusion and consumption parameters as a function of cell density.

	**Cell density (10**^****12****^ **cells · m**^****−3****^**)**		
**Estimated parameter**	**0.5**	**1**	**5**
**D** **(m**^**2**^** · s**^**−1**^**)**	1.2 · 10^−9^	1.1 · 10^−9^	0.9 · 10^−9^
σ_*D*_ (m^2^ · s^−1^)	1.2 · 10^−12^	1.2 · 10^−12^	6.6 · 10^−12^
*CV*_*D*_ (%)	0.10	0.11	0.73
**K**_**m**_ **(mol · m**^**−3**^**)**	4.1 · 10^−3^	6.2 · 10^−3^	2.8 · 10^−2^
σ_*K*_*m*__ (mol · m^−3^)	6.1 · 10^−5^	6.5 · 10^−5^	8.0 · 10^−4^
*CV*_*K*_*m*__ (%)	1.49	1.05	2.86
**V**_***max***_ **(mol · m**^**−3**^** · s**^**−1**^**)**	6.1 · 10^−5^	6.9 · 10^−5^	1.3 · 10^−4^
σ_*V*_***max***__ (mol · m^−3^ · s^−1^)	1.4 · 10^−7^	1.5 · 10^−7^	1.7 · 10^−6^
*CV*_*V*_***max***__ (%)	0.23	0.22	1.31
***sOCR*** **(amol · cell**^**−1**^** · s**^**−1**^**)**	122	69	26
σ_*sOCR*_ (amol · cell^−1^ · s^−1^)	0.28	0.15	0.34
ϵ_*sOCR*_ (%)	0.23	0.22	1.31

*In table, σ_p_ and CV_p_ denote the standard deviation and the coefficient of variation obtained for the estimated parameter p, respectively*.

Notably, the *CV* values were very low for all the 3 estimated parameters (i.e., *D*, *K*_*m*_, and *V*_max_), confirming the goodness of fit and indicating that the Michaelis-Menten law accurately describes the oxygen consumption kinetics in these constructs for each cell density. However, the reaction parameters are not constants but depend on the cell density.

### Average vs. Single Cell Oxygen Consumption Rate (*OCR* vs. *sOCR*)

**Figure 4:**
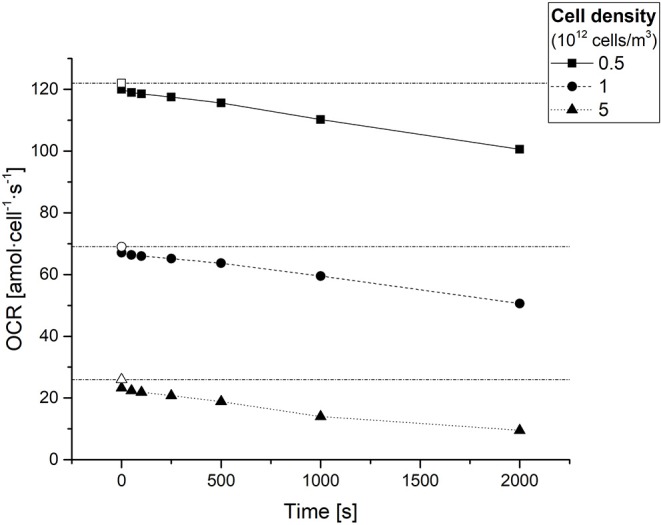
Average cellular oxygen consumption rate (*OCR*) in hepatocyte laden gels as a function of time for different cell densities. Empty symbols on the y-axis represent the respective (time-independent) *sOCRs* estimated for different cell densities. The dash-dotted horizontal lines serve only as a guide to the eye for identifying the three *sOCR* values.

After estimating the values of *D*, *K*_*m*_, and *V*_max_ for each condition, the average *OCR* of the 3D constructs at different timepoints was obtained using numerical methods through Equation 5. Figure 4 shows the average *OCR* as a function of cell density, computed at different experimental times (*t* = 0, 50, 100, 250, 500, 1,000, and 2,000 s).

The *OCR* in the constructs decreases with time and depends on the cell density, as expected from the decrease in oxygen concentration over time and the decrease in *sOCR* with increasing cell density, respectively. Notably, at time zero, when the oxygen concentration throughout the construct is maximum, the *OCR* approaches (but does not equal) the *sOCR*.

## Discussion

In this paper we describe a study to accurately evaluate the OCR in a 3D construct without assuming any prior knowledge of the model parameters (*D, V*_*max*_*, K*_*m*_). Ehsan and George (2013) report an experimental set up and theoretical approach similar to ours. The authors assume literature values for all the constants and then directly solve for oxygen profiles using finite element software. Here, as far as we know, for the first time the Michaelis- Menten constants are treated as unknowns and an inverse (iterative) problem approach is used for parameter estimation.

Only a small decrease in the oxygen diffusion coefficient throughout the hydrogel construct (*D*) was observed with increasing cell density, as expected due to the very low volume fraction occupied by cells (<1%). Notably, the expected increase in *V*_max_ with increasing cell density was concomitant with a decrease in *sOCR* and an increase in *K*_*m*_, both of which suggest the presence of *cooperative* behavior in the cell oxygen consumption characteristics, which are dependent on cell density. This *cooperative* behavior is not accounted for in the relatively simple Michaelis-Menten model, which describes cell oxygen consumption kinetics as an *adaptive* behavior depending only on the local O_2_ concentration perceived by cells. All the other parameters (i.e., *K*_*m*_ and *sOCR*) are considered as constants depending on the specific cell type, but not on their density. The results indicate that cells somehow sense the increase in number and consequently not only reduce their maximal oxygen consumption rate (*sOCR*), but also their affinity toward oxygen (i.e., decrease the efficiency with which oxygen is captured), as indicated by the increase in *K*_*m*_. This is also reflected in the fact that the average *OCR* decreases with increasing cell density as predicted by Ahluwalia (2017). In addition, for a given cell density the (average) *OCR* decreases with time. If we assume that the cell number is constant (i.e., no cell proliferation or death**—**reasonable in the short time frame of the 1 h experiments), then clearly the *OCR* is also a function of oxygen concentration. This may explain why measured *OCRs* vary so widely in the literature: slight changes in media heights, even in monolayer cultures, can cause large changes in the oxygen concentration perceived by cells.

Beyond these considerations, one should note that in a typical cell culture experiment, oxygen transport and consumption is governed by two Fick's laws (Mattei et al., 2014): (i) one for the “culture medium” domain (Equation 15, subscript *m*) where oxygen is transported by diffusion (plus convection in the presence of a velocity field ***u*** as in bioreactors, or micro-fluidic devices), and (ii) one for the “cell construct” compartment (Equation 16, subscript *c*), where oxygen is generally transported by passive diffusion and consumed at a rate *R*, which is typically modeled according to the Michaelis-Menten kinetics, as outlined in the introduction. The internal boundary between culture medium and cell construct domain is characterized by continuity of oxygen concentration and flux.

(15) $$\begin{array}{lll} \frac{\partial c_{m}}{\partial t} & = & \nabla \;\cdot \;\left(D_{m}\nabla c_{m}\right)-u\;\cdot \;\nabla c_{m} \end{array}$$

(16) $$\begin{array}{lll} \frac{\partial c_{c}}{\partial t} & = & \nabla \;\cdot \;\left(D_{c}\nabla c_{c}\right)-R \end{array}$$

Often, the average *OCR* is derived by measuring the first time-derivative of the oxygen concentration decrease in the “culture medium” compartment (i.e., ∂*c*_*m*_/∂*t*) and dividing it by the cell density (or number of cells divided by the volume of media) (Patzer, 2004; Russell et al., 2017), neglecting oxygen gradients in the culture medium and assuming that the rate at which oxygen disappears from the medium is equal to the rate of oxygen utilization by the cell construct.

The assumption that $\frac{\partial c_{m}}{\partial t}=R$ can be reasonable for 2D cultures, since *de facto* the cell monolayer represents a boundary of the culture medium compartment, thus the quantity of oxygen disappearing from the media over time is equal to that consumed by cells. However, the same cannot be said in the presence of 3D cultures, where oxygen diffusion throughout the construct cannot be neglected. For a construct in contact with a well-defined medium domain, the rate of decrease in oxygen concentration as measured from the culture medium side is a consequence of both its diffusion [i.e., ∇ · (*D*_*c*_∇*c*_*c*_)] and consumption (*R*) in the 3D cellular domain. Hence, the *average OCR* cannot be directly derived as ∂*c*_*m*_/∂*t* divided by ρ_*cell*_ as doing so would likely lead to an overestimation of the cellular uptake rate. Approaches in which the boundary conditions are controllable and known and in which the oxygen consumption is measured within the construct, as described herein are thus required to get meaningful values of *average OCR* and *sOCR*.

Since the oxygen consumption kinetics depends (among others) on the available O_2_ concentration, it is of interest to consider the following definition of the Thiele modulus (Equation 17), which conveniently includes the transition from a zero- to a first-order consumption kinetics as the O_2_ concentration decreases and returns a time-dependent parameter, differently than the classical Thiele modulus which is a constant (Equation 10) (Fink et al., 1973):

(17) $$\begin{array}{l} \phi^{*}\left(t\right)=H\sqrt{\frac{sOCR\;\cdot \;\rho_{cell}}{D\;\cdot \;\left(K_{m}+C_{av}\left(t\right)\right)}} \end{array}$$

In the latter equation, *C*_*av*_(*t*) denotes the time-dependent volumetric average of the local oxygen concentration *C*(*z, t*), defined as follows for the cylindrically symmetric problem investigated in this work:

(18) $$\begin{array}{l} C_{av}\left(t\right)=\frac{1}{H}\int C\left(z,\;t\right)\partial z \end{array}$$

**Figure 5:**
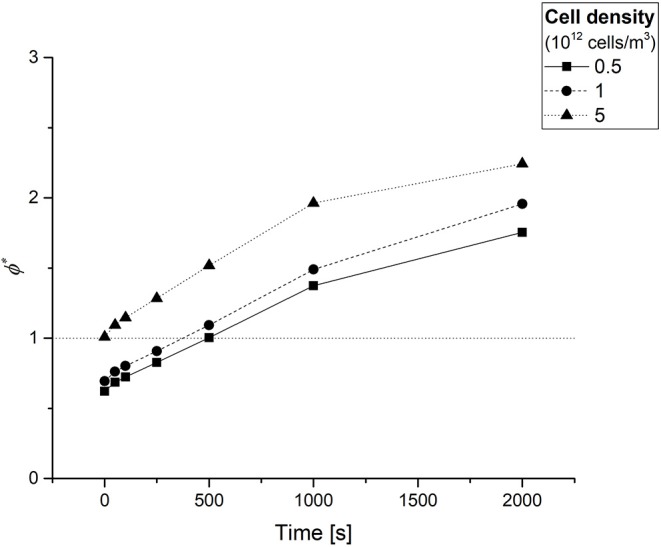
The time dependent Thiele modulus ϕ* obtained at different experimental times for each of the three cell densities investigated. The dotted horizontal line serves only as a guide to the eye for identifying the instant at which ϕ* = 1.

Figure 5 shows the ϕ^*^ temporal profiles computed at different experimental times (*t* = 0, 50, 100, 250, 500, 1,000, and 2,000 s) for each of the three cell densities under study. At each time point, the ϕ^*^ increases with cell density, as one would expect because of the increasing oxygen demand. At time zero, the oxygen supply meets the cell demand for each of the cell densities investigated, as indicated by ϕ^*^ ≤ 1. Afterwards, this parameter increases monotonically over time. Notably, the instant at which ϕ^*^ = 1 (corresponding to the point where oxygen consumption rate equates the oxygen diffusion rate) is reached more rapidly in denser cell-laden hydrogel constructs. Beyond this point, oxygen diffusion becomes the limiting factor in the diffusion-reaction processes occurring within the cellularized hydrogel constructs (Mattei et al., 2014).

## Conclusions

The oxygen concentration perceived by cells conditions their function and behavior. Therefore, the capacity to monitor oxygen in a culture and to quantify the cell oxygen consumption kinetics is critical for the development of tissue engineered products. However, the measurement of the average cellular consumption rate in 3D constructs, known as the OCR, is difficult because of the lack of tools for the precise quantification of oxygen concentration in space and time in tissues. Our results show that the OCR is neither a constant for a given cell type nor is it constant in a given tissue or tissue construct. Moreover, for given construct dimensions, the Michaelis-Menten constant and the value of *V*_*max*_ as well as the oxygen diffusion coefficient depend on the number of cells in the tissue or scaffold volume. Thus, consumption parameters should not be assumed as literature constants. Given the importance of oxygen consumption in tissues, this study paves the way for a more meaningful interpretation of the significance of OCR, the refinement of analytical techniques and better control of resource supply to 3D *in-vitro* cell culture systems. The method described in this paper can be used to determine the OCR as a function of cell density in any 3D construct seeded with any cell type. Moreover, it can be also used to calculate the OCR at discrete time points provided the cell density is accurately known at the time points of interest.

## Data Availability Statement

The datasets used and/or analyzed during the current study are available from the corresponding author upon reasonable request.

## Author Contributions

GM and AA conceived and designed the work. FI, AC, and CM performed the experiments and acquired data and images, GM, FI, CM, VP, and AA interpreted and analyzed the data. GM and AA drafted the work. All the authors have approved the submitted version and have agreed both to be personally accountable for their own contributions and to ensure that questions related to the accuracy or integrity of any part of the work, even ones in which the author was not personally involved, are appropriately investigated, resolved, and the resolution documented in the literature.

### Conflict of Interest

The authors declare that the research was conducted in the absence of any commercial or financial relationships that could be construed as a potential conflict of interest.

## References

[B1] Ahluwalia A. (2017). Allometric scaling *in-vitro*. Sci Rep. 7:42113. 10.1038/srep42113 28169362 PMC5294453

[B2] Ahluwalia A. Misto A. Vozzi F. Magliaro C. Mattei G. Marescotti M. C.. (2018). Systemic and vascular inflammation in an *in-vitro* model of central obesity. PLoS ONE 13:e0192824. 10.1371/journal.pone.0192824 29438401 PMC5811040

[B3] Balis U. J. Behnia K. Dwarakanath B. Bhatia S. N. Sullivan S. J. Yarmush M. L.. (1999). Oxygen consumption characteristics of porcine hepatocytes. Metab. Eng. 1, 49–62. 10.1006/mben.1998.0105 10935754

[B4] Brown D. A. MacLellan W. R. Laks H. Dunn J. C. Wu B. M. Beygui R. E. (2007). Analysis of oxygen transport in a diffusion-limited model of engineered heart tissue. Biotechnol. Bioeng. 97, 962–975. 10.1002/bit.21295 17195988

[B5] Cheema U. Brown R. A. Alp B. MacRobert A. J. (2008). Spatially defined oxygen gradients and vascular endothelial growth factor expression in an engineered 3D cell model. Cell Mol. Life Sci. 65, 177–186. 10.1007/s00018-007-7356-8 17994289 PMC11131842

[B6] Cheema U. Rong Z. Kirresh O. MacRobert A. J. Vadgama P. Brown R. A. (2012). Oxygen diffusion through collagen scaffolds at defined densities: implications for cell survival in tissue models. J. Tissue Eng. Regen. Med. 6, 77–84. 10.1002/term.402 21312340

[B7] Cheng G. Markenscoff P. Zygourakis K. (2009). A 3D hybrid model for tissue growth: the interplay between cell population and mass transport dynamics. Biophys. J. 97, 401–414. 10.1016/j.bpj.2009.03.067 19619455 PMC2711338

[B8] Cho C. H. Park J. Nagrath D. Tilles A. W. Berthiaume F. Toner M.. (2007). Oxygen uptake rates and liver-specific functions of hepatocyte and 3T3 fibroblast co-cultures. Biotechnol. Bioeng. 97, 188–199. 10.1002/bit.21225 17054120

[B9] Curcio E. Macchiarini P. De Bartolo L. (2010). Oxygen mass transfer in a human tissue-engineered trachea. Biomaterials 31, 5131–5136. 10.1016/j.biomaterials.2010.03.013 20378162

[B10] Demol J. Lambrechts D. Geris L. Schrooten J. Van Oosterwyck H. (2011). Towards a quantitative understanding of oxygen tension and cell density evolution in fibrin hydrogels. Biomaterials 32, 107–118. 10.1016/j.biomaterials.2010.08.093 20880579

[B11] Ehsan S. M. George S. C. (2013). Nonsteady state oxygen transport in engineered tissue: implications for design. Tissue Eng. Part A 19, 1433–1442. 10.1089/ten.tea.2012.0587 23350630 PMC3638538

[B12] Fink D. J. Na T. Schultz J. S. (1973). Effectiveness factor calculations for immobilized enzyme catalysts. Biotechnol. Bioeng. 15, 879–888. 10.1002/bit.260150505

[B13] Folkman J. Moscona A. (1978). Role of cell shape in growth control. Nature 273, 345–349. 10.1038/273345a0 661946

[B14] Freshney R. I. (2015). Culture of Animal Cells: A Manual of Basic Technique. New York, NY: Wiley.

[B15] Glazier D. (2015). Body-mass scaling of metabolic rate: what are the relative roles of cellular versus systemic effects? Biology 4, 187–199. 10.3390/biology4010187 25808601 PMC4381225

[B16] Helmlinger G. Yuan F. Dellian M. Jain R. K. (1997). Interstitial pH and pO2 gradients in solid tumors *in vivo*: high-resolution measurements reveal a lack of correlation. Nat. Med. 3, 177–182. 10.1038/nm0297-177 9018236

[B17] Lee M. Dunn J. C. Y. Wu B. M. (2005). Scaffold fabrication by indirect three-dimensional printing. Biomaterials 26, 4281–4289. 10.1016/j.biomaterials.2004.10.040 15683652

[B18] Lewis M. C. MacArthur B. D. Malda J. Pettet G. Please C. P. (2005). Heterogeneous proliferation within engineered cartilaginous tissue: the role of oxygen tension. Biotechnol. Bioeng. 91, 607–615. 10.1002/bit.20508 16025534

[B19] Mac Gabhann F. Ji J. W. Popel A. S. (2007). VEGF gradients, receptor activation, and sprout guidance in resting and exercising skeletal muscle. J. Appl. Physiol. 102, 722–734. 10.1152/japplphysiol.00800.2006 17038488

[B20] Magliaro C. Rinaldo A. Ahluwalia A. (2019). Allometric Scaling of physiologically-relevant organoids. Sci. Rep. 4:559682 10.1101/559682 PMC6695443 31417119

[B21] Malda J. Rouwkema J. Martens D. E. Le Comte E. P. Kooy F. K. Tramper J.. (2004). Oxygen gradients in tissue-engineered PEGT/PBT cartilaginous constructs: measurement and modeling. Biotechnol. Bioeng. 86, 9–18. 10.1002/bit.20038 15007836

[B22] Martin Y. Vermette P. (2005). Bioreactors for tissue mass culture: design, characterization, and recent advances. Biomaterials 26, 7481–7503. 10.1016/j.biomaterials.2005.05.057 16023202

[B23] Mattei G. Giusti S. Ahluwalia A. (2014). Design criteria for generating physiologically relevant *in vitro* models in bioreactors. Processes 2, 548–569. 10.3390/pr2030548

[B24] Mattei G. Magliaro C. Giusti S. Ramachandran S. D. Heinz S. Braspenning J.. (2017a). On the adhesion-cohesion balance and oxygen consumption characteristics of liver organoids. PLoS ONE 12:e0173206. 10.1371/journal.pone.0173206 28267799 PMC5340403

[B25] Mattei G. Magliaro C. Pirone A. Ahluwalia A. (2017b). Decellularized human liver is too heterogeneous for designing a generic extracellular matrix mimic hepatic scaffold. Artif. Organs 41, E347–E355. 10.1111/aor.12925 28543403

[B26] Mattei G. Magliaro C. Pirone A. Ahluwalia A. (2018). Bioinspired liver scaffold design criteria. Organogenesis 14, 129–146. 10.1080/15476278.2018.1505137 30156955 PMC6300109

[B27] Nyberg S. L. Remmel R. P. Mann H. J. Peshwa M. V. Hu W. S. Cerra F. B. (1994). Primary hepatocytes outperform Hep G2 cells as the source of biotransformation functions in a bioartificial liver. Ann. Surg. 220, 59–67. 8024360 PMC1234288

[B28] Nyberg S. L. Shirabe K. Peshwa M. V. Sielaff T. D. Crotty P. L. Mann H. J.. (1993). Extracorporeal application of a gel-entrapment, bioartificial liver: demonstration of drug metabolism and other biochemical functions. Cell Transplant. 2, 441–452. 10.1177/096368979300200602 8167929

[B29] Patzer J. F. (2004). Oxygen consumption in a hollow fiber bioartificial liver–revisited. Artif. Organs 28, 83–98. 10.1111/j.1525-1594.2004.07150.x 14720293

[B30] Radisic M. Malda J. Epping E. Geng W. Langer R. Vunjak-Novakovic G. (2006). Oxygen gradients correlate with cell density and cell viability in engineered cardiac tissue. Biotechnol. Bioeng. 93, 332–343. 10.1002/bit.20722 16270298

[B31] Rivron N. C. Liu J, J. Rouwkema J. de Boer J. van Blitterswijk C. A. (2008). Engineering vascularised tissues *in vitro*. Eur. Cell Mater. 15, 27–40. 10.22203/eCM.v015a03 18288631

[B32] Russell S. Wojtkowiak J. Neilson A. Gillies R. J. (2017). Metabolic Profiling of healthy and cancerous tissues in 2D and 3D. Sci. Rep. 7:15285. 10.1038/s41598-017-15325-5 29127321 PMC5681543

[B33] Shatford R. A. Nyberg S. L. Meier S. J. White J. G. Payne W. D. Hu W. S.. (1992). Hepatocyte function in a hollow fiber bioreactor: a potential bioartificial liver. J. Surg. Res. 53, 549–557. 10.1016/0022-4804(92)90253-V 1494286

[B34] Sielaff T. D. Nyberg S. L. Rollins M. D. Hu M. Y. Amiot B. Lee A.. (1997). Characterization of the three-compartment gel-entrapment porcine hepatocyte bioartificial liver. Cell Biol. Toxicol. 13, 357–364. 10.1023/A:1007499727772 9298256

[B35] Smith M. D. Smirthwaite A. D. Cairns D. E. Cousins R. B. Gaylor J. D. (1996). Techniques for measurement of oxygen consumption rates of hepatocytes during attachment and post-attachment. Int. J. Artif. Organs 19, 36–44. 10.1177/039139889601900106 8641817

[B36] Tremper K. K. Barker S. J. Blatt D. H. Wender R. H. (1986). Effects of anesthetic agents on the drift of a transcutaneous oxygen tension sensor. J. Clin. Monit. 2, 234–236. 10.1007/BF02851171 3783195

[B37] Wagner B. A. Venkataraman S. Buettner G. R. (2011). The rate of oxygen utilization by cells. Free Radic. Biol. Med. 51, 700–712. 10.1016/j.freeradbiomed.2011.05.024 21664270 PMC3147247

[B38] Zhou S. Cui Z. Urban J. P. G. (2008). Nutrient gradients in engineered cartilage: metabolic kinetics measurement and mass transfer modeling. Biotechnol. Bioeng. 101, 408–421. 10.1002/bit.21887 18727036

